# 

**Published:** 1884

**Authors:** 


### COUGH

AND

### EXPECTORATION

### A REPERTORIAL INDEX OF THEIR SYMPTOMS

EDITED BY

E. JENNINGS LEE, M. D.,

ASSISTED BY

GEORGE H. CLARK, M. D.

PRINTED AS A SUPPLEMENT TO

THE HOMŒOPATHIC PHYSICIAN

1884

